# Rosiglitazone may reduce non-melanoma skin cancer risk in Taiwanese

**DOI:** 10.1186/s12885-015-1057-8

**Published:** 2015-02-06

**Authors:** Chin-Hsiao Tseng

**Affiliations:** 1 Department of Internal Medicine, National Taiwan University College of Medicine, No. 7 Chung-Shan South Road, Taipei, (100) Taiwan; 2 Division of Endocrinology and Metabolism, Department of Internal Medicine, National Taiwan University Hospital, Taipei, Taiwan; 3 Division of Environmental Health and Occupational Medicine of the National Health Research Institutes, Taipei, Taiwan

**Keywords:** Diabetes mellitus, Non-melanoma skin cancer, Rosiglitazone, Taiwan

## Abstract

**Background:**

Whether rosiglitazone may affect the risk of non-melanoma skin cancer (NMSC) has not been investigated.

**Methods:**

The reimbursement databases of all Taiwanese diabetic patients from 1996 to 2009 were retrieved from the National Health Insurance. An entry date was set at 1 January 2006 and a total of 886418 patients with type 2 diabetes were followed up for NMSC incidence until the end of 2009. Incidences for ever-users, never-users and subgroups of rosiglitazone exposure (using tertile cutoffs of duration of therapy and cumulative dose) were calculated and hazard ratios estimated by Cox regression. Additional models were created as sensitivity analyses.

**Results:**

There were 103097 ever-users and 783321 never-users, respective numbers of incident NMSC 250 (0.24%) and 2084 (0.27%), and respective incidence 68.90 and 76.77 per 100000 person-years. Although the overall hazard ratio was not significant in the unadjusted, age-sex-adjusted or fully adjusted model, the risk was significantly lower in the third tertile of duration of therapy and cumulative dose, with significant *P* for trends. The fully adjusted hazard ratio (95% confidence interval) for a duration of therapy >13.77 months and a cumulative dose of >1752 mg was 0.723 (0.566, 0.923) and 0.783 (0.618, 0.993), respectively. The findings were supported by various sensitivity analyses.

**Conclusions:**

Rosiglitazone may reduce the risk of NMSC, but further confirmation is required.

## Background

All three isotypes of peroxisome proliferator-activator receptors (PPARs), i.e., PPARα, PPARβ/δ and PPARγ, are expressed in human skin keratinocytes [1,2]. They play important roles in skin barrier permeability, proliferation inhibition, differentiation promotion, immune regulation, and sebum production [1,3].

PPARβ/δ is the most predominant isotype in human keratinocytes, and it is upregulated during wound healing [1]. PPARα is closely related to lipid metabolism; and plays an important role in skin barrier development [1]. PPARγ activation has been shown to inhibit proliferation, promote differentiation and induce apoptosis in various malignant tissues; and its agonists have been used as therapeutic agents for psoriasis, a benign skin disease characterized by epidermal hyperplasia [1,4-6].

The use of PPAR agonists or antagonists in the treatment of many skin diseases including acne vulgaris, psoriasis, benign skin tumors and skin cancer is of clinical importance but still awaits in-depth investigation [2]. Whether PPARγ has a role in the prevention or treatment of skin cancer is under debate [2]. Reduced PPARγ activity is noted in mice susceptible to skin cancer induced by dimethylbenz[a]anthracene [7], but activation of PPARγ with rosiglitazone treatment does not prevent the development of skin tumors induced by ultraviolet light or chemical such as dimethylbenz[a]anthracene/12-O-tetradecanoylphorbol-13-acetate in mice [8]. However, a later study by the same group did show that rosiglitazone may reduce the occurrence of skin cancer in transgenic mice overexpressing insulin-like growth factor 1 (IGF1) [9].

To our knowledge, there is only one paper evaluating the risk of non-melanoma skin cancer (NMSC) in patients who had used thiazolidinediones (a class of PPARγ agonists used for glycemic control in patients with type 2 diabetes mellitus) by using the General Practice Research Database in the United Kingdoms [10]. The investigators reported a lack of association without specifying the various thiazolidinediones used by the patients.

In an early study conducted in keratinocytes in monolayer culture and in human whole skin organ culture, rosiglitazone (one of the thiazolidinediones used in clinical practice) inhibits proliferation, motility, and matrix metalloproteinase production in keratinocytes more effectively than the other PPARγ agonist pioglitazone does [11]. In consideration of the better performance of rosiglitazone on the inhibition of keratinocyte proliferation [11], and the recently reported potential risk of bladder cancer related to pioglitazone [12-14], the present study aimed at evaluating the association between rosiglitazone use and NMSC in Taiwanese patients with type 2 diabetes mellitus by using the reimbursement databases of the National Health Insurance (NHI).

## Methods

The study was approved by an ethic review board of the National Health Research Institutes with registered approval number 99274.

Since March 1995, a compulsory and universal system of health insurance (the so-called NHI) was implemented in Taiwan. All contracted medical institutes must submit computerized and standard claim documents for reimbursement. More than 99% of citizens are enrolled in the NHI, and >98% of the hospitals nationwide are under contract with the NHI. The average number of annual physician visits in Taiwan is one of the highest around the world, at approximately 15 visits per year per capita in 2009.

The National Health Research Institutes is the only organization approved, as per local regulations, for handling the NHI reimbursement databases for academic research. The databases contain detailed records on every visit for each patient, including outpatient visits, emergency department visits and hospital admission. The databases also include principal and secondary diagnostic codes, prescription orders, and claimed expenses.

The identification information of the individuals was scrambled for the protection of privacy. Diabetes was coded 250.XX and NMSC 173, based on the *International Classification of Diseases, Ninth Revision, Clinical Modification* (ICD-9-CM).

The databases of all patients who had been diagnosed with diabetes and under treatment with either oral anti-diabetic agents or insulin during the period of 1996–2009 from the whole nation, and who remained enrolled in the NHI after 2006 (*n* = 1446414) were first retrieved. A total of 235287 patients who had ever been treated with pioglitazone were then excluded to avoid the possible contamination by its use because it may increase the risk of some cancer like bladder cancer [15]. The selected entry date was 1 January 2006. After excluding patients who had a diagnosis of diabetes after the year 2006 (*n* = 342351), patients who held a Severe Morbidity Card as having type 1 diabetes (*n* = 7120, in Taiwan, patients with type 1 diabetes were issued a so-called “Severe Morbidity Card” after certified diagnosis and they were waived for much of the co-payments), patients having a diagnosis of NMSC before 2006 (*n* = 6297), those who died (*n* = 96320) or withdrew from the NHI (*n* = 12502) before entry date, duplicated identification number (*n* = 106), and unclear information on date of birth or sex (*n* = 5123), a total of 886418 patients with a diagnosis of type 2 diabetes mellitus and under therapy with oral anti-diabetic agents (except pioglitazone) or insulin were recruited.

Those who had ever been prescribed with rosiglitazone before entry date were defined as ever-users; and never-users were defined as those who had never been prescribed with rosiglitazone before entry date. To evaluate whether a dose–response relationship could be seen between rosiglitazone and NMSC, the tertile cutoffs for duration of therapy in months and cumulative dose in mg were calculated from the databases and used for analyses.

An entry date at the beginning of 2006 was used based on the following reasons: 1) Because rosiglitazone was marketed in 2001 in Taiwan, this entry date, being in the middle of the marketing date of rosiglitazone in Taiwan and the ending date of the available NHI databases in 2009, provided a longest exposure of 4 to 5 years at entry and at the same time a longest follow-up duration of 4 years; and 2) The issue of bladder cancer associated with pioglitazone noted in the PROspective pioglitAzone Clinical Trial In macroVascular Events (PROactive) was published in 2005 [16], and in 2007, the safety of rosiglitazone has been challenged with a risk of acute myocardial infarction [17]. These had caused tremendous prescription behavior changes in the physicians to withdraw thiazolidinediones including rosiglitazone and pioglitazone (troglitazone has not been marketed in Taiwan) and the patients might have stopped taking the drugs even if they were prescribed after the year 2006. Therefore, the use of a later entry date would make the estimation of the duration of therapy and cumulative dose of rosiglitazone less reliable. In addition, this would also shorten the follow-up duration for cancer incidence.

All comorbidities and covariates were determined as a status/diagnosis before the entry date. The ICD-9-CM codes for the comorbidities were [18-21]: nephropathy 580–589, hypertension 401–405, chronic obstructive pulmonary disease (a surrogate for smoking) 490–496, cerebrovascular disease 430–438, ischemic heart disease 410–414, peripheral arterial disease 250.7, 785.4, 443.81 and 440–448, eye disease 250.5, 362.0, 369, 366.41 and 365.44, obesity 278, dyslipidemia 272.0-272.4, and cancer other than NMSC 140–208 (excluding 173). Medications included sulfonylurea, metformin, insulin, and acarbose. Baseline characteristics between ever-users and never-users of rosiglitazone were compared by Chi-square test.

The incidence density of NMSC was calculated for ever-users and never-users and for different subgroups of exposure. Chi-square test was used to compare the distribution of incident cases of NMSC in ever-users versus never-users and among the different subgroups of dose–response parameters. The numerator for the incidence was the number of patients with incident NMSC during the 4-year follow-up, and the denominator was the person-years of follow-up. For ever-users, the follow-up duration was either censored at the date of NMSC diagnosis or at the date of the last record of the available reimbursement databases in individuals without incident NMSC. For never-users, the follow-up was censored at the date of rosiglitazone initiation or NMSC diagnosis or the last reimbursement record, depending on whichever occurring first. This ensured no exposure to rosiglitazone throughout the whole follow-up period until censor in the referent group of never-users.

Cox proportional hazards regression was performed to estimate the hazard ratios for NMSC for ever-users versus never-users, and for the various subgroups of dose–response parameters. The following three models were created: 1) unadjusted; 2) adjusted for age and sex; and 3) adjusted for all variables compared previously as baseline characteristics between ever-users and never-users.

The following fully adjusted models were also created as sensitivity analyses: 1) resetting the entry date to 1 January 2005; 2) deleting patients who developed NMSC within 3 months of follow-up; 3) excluding patients with a history of any cancer before 2006; 4) including pioglitazone users in the analyses; 5) using a time-dependent approach; and 6) excluding never-users of rosiglitazone and conducting the analyses only among rosiglitazone ever-users by comparing the second and third tertiles of exposure versus the first tertiles as referents.

Analyses were conducted using SAS statistical software, version 9.3 (SAS Institute, Cary, NC). *P* < 0.05 was considered statistically significant.

## Results

Table 1 compares the baseline characteristics between ever-users (*n* = 103097) and never-users (*n* = 783321) of rosiglitazone. All of the variables differed significantly between the two groups. Ever-users are characterized by older age distribution, higher proportion with a diabetes duration ≥5 years, higher proportions of all comorbidities and other cancer, higher proportions of using other medications.

**Table 1:** **Baseline characteristics between never-users and ever-users of rosiglitazone**

Variables	Never-users		Ever-users		*P*
	*n*	%	*n*	%	
*n* = 886418	783321		103097		
Age (years)					
<40	33874	4.32	3269	3.17	<0.0001
40-49	102887	13.13	11623	11.27	
50-59	199819	25.51	27204	26.39	
60-69	200241	25.56	29608	28.72	
≥70	246500	31.47	31393	30.45	
Sex (men)	399333	50.98	49282	47.80	<0.0001
Diabetes duration (years)					
<1	70381	8.98	1434	1.39	<0.0001
1-3	133250	17.01	7184	6.97	
3-5	123610	15.78	11094	10.76	
≥5	456080	58.22	83385	80.88	
Hypertension	443728	56.65	77740	75.40	<0.0001
Chronic obstructive pulmonary disease	114018	14.56	24177	23.45	<0.0001
Cerebrovascular disease	116947	14.93	25453	24.69	<0.0001
Nephropathy	93744	11.97	23717	23.00	<0.0001
Ischemic heart disease	174225	22.24	40511	39.29	<0.0001
Peripheral arterial disease	89324	11.40	24510	23.77	<0.0001
Eye disease	63803	8.15	21179	20.54	<0.0001
Obesity	11423	1.46	1961	1.90	<0.0001
Dyslipidemia	371714	47.45	69015	66.94	<0.0001
Other cancer prior to baseline	93619	11.95	13058	12.67	<0.0001
Sulfonylurea	577003	73.66	99140	96.16	<0.0001
Metformin	512063	65.37	96850	93.94	<0.0001
Acarbose	80640	10.29	38971	37.80	<0.0001
Insulin	102359	13.07	39119	37.94	<0.0001

Table 2 shows the incidences of NMSC between ever-users and never-users of rosiglitazone, and among the different categories of the dose–response parameters for rosiglitazone exposure. The incidence rate in never-users and ever-users of rosiglitazone was 76.77 and 68.90 per 100000 person-years, respectively.

**Table 2:** **Incidence of non-melanoma skin cancer by rosiglitazone exposure at entry**

Rosiglitazone use	Case number	Incident skin cancer	%	Person-years	Incidence rate (per 100,000 person-years)
Never users	783321	2084	0.27	2714745.08	76.77
Ever users	103097	250	0.24	362846.42	68.90
*P* value			0.1653		
**Duration of therapy (months)**					
Never users	783321	2084	0.27	2714745.08	76.77
<3.73	33039	93	0.28	112752.67	82.48
3.73-13.77	35108	88	0.25	123278.50	71.38
>13.77	34950	69	0.20	126815.25	54.41
*P* value			0.0847		
**Cumulative dose (mg)**					
Never users	783321	2084	0.27	2714745.08	76.77
<448	32942	90	0.27	112711.50	79.85
448-1752	35078	86	0.25	123002.83	69.92
>1752	35077	74	0.21	127132.08	58.21
*P* value			0.2172		

Table 3 shows the hazard ratios with regards to different categories of rosiglitazone exposure. In the models evaluating the overall hazard ratios for ever-users versus never-users, the hazard ratios were not significant in all three models. However, in the models evaluating the dose–response exposure to rosiglitazone with regards to duration of therapy and cumulative dose, a significantly lower risk could be seen in the third tertiles and the trend analyses.

**Table 3:** **Rosiglitazone exposure at entry and hazard ratios for non-melanoma skin cancer**

Rosiglitazone use	Unadjusted			Age-sex-adjusted			Fully adjusted*		
	HR	95% CI	*P*	HR	95% CI	*P*	HR	95% CI	*P*
Ever users	0.882	(0.773, 1.005)	0.0602	0.908	(0.796, 1.036)	0.1513	0.927	(0.807, 1.066)	0.2865
**Duration of therapy (months)**									
<3.73	1.059	(0.861, 1.304)	0.5862	1.109	(0.901, 1.365)	0.3295	1.119	(0.905, 1.383)	0.2985
3.73-13.77	0.903	(0.730, 1.118)	0.3507	0.941	(0.760, 1.165)	0.5744	0.961	(0.773, 1.196)	0.7243
>13.77	0.703	(0.553, 0.893)	0.0039	0.705	(0.555, 0.896)	0.0043	0.723	(0.566, 0.923)	0.0094
P-trend			0.0072			0.0171			0.0386
**Cumulative dose (mg)**									
<448	1.025	(0.830, 1.265)	0.8221	1.070	(0.867, 1.322)	0.5276	1.081	(0.872, 1.341)	0.4773
448-1752	0.885	(0.713, 1.098)	0.2675	0.912	(0.735, 1.132)	0.4033	0.931	(0.747, 1.161)	0.5270
>1752	0.752	(0.596, 0.948)	0.0157	0.764	(0.606, 0.963)	0.0228	0.783	(0.618, 0.993)	0.0436
P-trend			0.0139			0.0337			0.0743

Referent group: never-users of rosiglitazone; HR: hazard ratio, CI: confidence intervals.

*Adjusted for all variables in Table 1.

The sensitivity analyses are shown in Table 4. Generally speaking, the results were consistent with the full models in the original analyses (Table 3). Although the overall hazard ratios were not significant, the third tertiles of rosiglitazone exposure were generally associated with a significantly lower risk of NMSC. In the time-dependent analyses, an approximately 30% higher risk was observed for the first tertiles of duration of therapy and cumulative dose, but the third tertiles remained significantly associated with a lower risk. In the analyses comparing the second and third tertiles of rosiglitazone exposure versus the first tertiles as referent by including only rosiglitazone ever-users, the hazard ratio (95% confidence interval) for the second and third tertile of duration of therapy was 0.850 (0.634, 1.140) (P = 0.2777) and 0.639 (0.465, 0.876) (P = 0.0054), respectively (P-trend = 0.0054); and was 0.852 (0.633, 1.148) and 0.712 (0.521, 0.973), respectively, for cumulative dose (P-trend = 0.0327).

**Table 4:** **Sensitivity analyses estimating hazard ratios for non-melanoma skin cancer associated with rosiglitazone exposure**

Rosiglitazone use	Setting entry date to 1 January 2005			Deleting patients who developed skin cancer within 3 months of follow-up			Excluding patients with a history of any cancer before 2006			Including pioglitazone users in the analyses			Time-dependent models		
	HR	95% CI	*P*	HR	95% CI	*P*	HR	95% CI	*P*	HR	95% CI	*P*	HR	95% CI	*P*
Ever users	0.986	(0.882, 1.102)	0.7977	0.933	(0.809, 1.075)	0.3368	0.951	(0.834, 1.083)	0.4469	0.938	(0.835, 1.055)	0.2863	0.909	(0.785, 1.051)	0.1981
**Duration of therapy (months)**															
<3.73	1.177	(0.975, 1.421)	0.0903	1.119	(0.900, 1.392)	0.3106	1.055	(0.855, 1.302)	0.6199	1.114	(0.930, 1.336)	0.2411	1.287	(1.050, 1.579)	0.0153
3.73-13.77	1.112	(0.939, 1.318)	0.2189	0.989	(0.792, 1.234)	0.9194	1.096	(0.906, 1.325)	0.3452	1.025	(0.862, 1.219)	0.7781	1.059	(0.851, 1.317)	0.6099
>13.77	0.787	(0.662, 0.935)	0.0065	0.713	(0.554, 0.918)	0.0087	0.737	(0.592, 0.916)	0.0060	0.718	(0.589, 0.874)	0.0010	0.583	(0.457, 0.740)	<0.0001
P-trend			0.1215			0.0484			0.0871			0.0186			0.0024
**Cumulative dose (mg)**															
<448	1.138	(0.939, 1.379)	0.1883	1.092	(0.876, 1.361)	0.4336	0.985	(0.793, 1.223)	0.8882	1.059	(0.880, 1.275)	0.5418	1.286	(1.030, 1.607)	0.0264
448-1752	1.099	(0.929, 1.301)	0.2722	0.923	(0.735, 1.159)	0.4892	1.105	(0.915, 1.334)	0.3015	1.011	(0.849, 1.203)	0.9047	0.983	(0.775, 1.247)	0.8876
>1752	0.815	(0.687, 0.966)	0.0186	0.798	(0.627, 1.016)	0.0672	0.780	(0.630, 0.965)	0.0224	0.773	(0.638, 0.936)	0.0084	0.614	(0.476, 0.792)	0.0002
P-trend			0.1839			0.0971			0.1685			0.0482			0.0041

Referent group: never-users of rosiglitazone; HR: hazard ratio, CI: confidence intervals.

In models excluding patients not treated with rosiglitazone and using the first tertile of exposure as referent, the hazard ratio (95% confidence interval) for the second and third tertile of duration of therapy was 0.850 (0.634, 1.140) (P = 0.2777) and 0.639 (0.465, 0.876) (P = 0.0054), respectively (P-trend = 0.0054); and was 0.852 (0.633, 1.148) and 0.712 (0.521, 0.973), respectively, for cumulative dose (P-trend = 0.0327).

## Discussion

This is the first observational cohort study evaluating the risk of NMSC associated with the specific use of rosiglitazone in patients with type 2 diabetes mellitus. The findings suggested a reduced risk in patients who had used the drug for more than 13 months or with a cumulative dose of greater than 1750 mg (Table 3). The results of such a promising effect provided rationale for further in-depth investigation on this drug in the prevention of NMSC.

The findings were consistent in various sensitivity analyses, except in the time-dependent models that showed a significantly higher risk in the first tertiles of rosiglitazone exposure (Table 4). In the analyses for the incidence of NMSC, patients in the first tertiles of rosiglitazone exposure also showed a slightly higher incidence than the referent groups of never-users of rosiglitazone (Table 2). This could result from residual confounding among users of a short duration (i.e., in the first tertiles of exposure) because rosiglitazone is always used as a second or third line drug for glucose lowering in clinical practice. At the inception of its use, patients are usually characterized by older age, a longer duration of diabetes (Table 1) and poorer glycemic control (data not available), which might themselves be associated with an increased risk of cancer development.

Exposure to ultraviolet sunlight may be the most important risk factor for skin cancer; and other risk factors may include family history, fair skin, light eyes, and immunosuppression [22]. It should be admitted that most of these potential confounders could not be adjusted for in the present study because of the lack of such information in the reimbursement databases. Therefore, the beneficial effect of rosiglitazone on the prevention of NMSC should better be confirmed in future studies considering the adjustment for these potential confounders.

Misclassification of NMSC might occur in the present study. However, such a probability was low because labeled diagnoses should be printed out in all prescriptions handed to the patients, and mislabeling of a cancer diagnosis would not be acceptable to the patients when they saw the diagnosis.

Because the present study recruited only patients with type 2 diabetes mellitus, the results should not be readily generalized to non-diabetic individuals. The animal studies conducted by He et al. have provided good reasons for this cautiousness. In ordinary mice treated with carcinogens, rosiglitazone does not reduce skin cancer risk [8], but rosiglitazone does reduce skin cancer risk in transgenic mice overexpressing IGF1 [9]. The authors explained that ultraviolet light and carcinogenic chemicals may activate some carcinogenic pathways enabling keratinocytes to bypass the IGF1 signaling; and that rosiglitazone may possibly inhibit the development of skin cancer through activation of AMP-activated protein kinase with subsequent inhibition of IGF1-induced mammalian target of rapamycin activity and phosphorylation of p70S6 kinase [9]. Patients with type 2 diabetes mellitus are at an increased risk of cancer and they are characterized by insulin resistance with hyperinsulinemia and hyperactivity in IGF1 signaling [23]. Therefore, more studies are required to clarify the usefulness of rosiglitazone on the prevention of skin cancer in individuals without diabetes mellitus.

This study has several strengths. Because the databases were derived from the whole population and they spanned the whole period from the beginning of the marketing of rosiglitazone from 2001 in Taiwan to the end of 2009, there was no concern of potential selection bias related to sampling error. The databases included all claim records on outpatient visits, emergency department visits and hospital admission, and we caught the diagnoses from all sources. Cancer is considered a severe morbidity by the NHI and most medical co-payments can be waived. Furthermore, there is a low drug cost-sharing required by the NHI and patients with certain conditions such as low-income household, veterans or patients with prescription refills for chronic disease are exempted from the drug cost-sharing. Therefore the detection rate of NMSC would not tend to differ among different social classes. The use of medical records also reduced the potential bias related to self-reporting.

The study limitations included a lack of actual measurement data for potential confounders such as sunlight exposure, occupation history, family history, lifestyle, diet, and genetic parameters. In addition, we did not have biochemical data for evaluating their impact. Another limitation is the lack of information on the histopathology, grading and staging of NMSC.

In summary, this study first reports a potential beneficial effect of the use of rosiglitazone on the prevention of NMSC in patients who have been using the medication for more than 13 months or with a cumulative dose of greater than 1750 mg.

## Conclusions

This large population-based retrospective cohort study in Taiwan suggests that prolonged use of rosiglitazone in patients with type 2 diabetes mellitus may be associated with a lower risk of NMSC. Exploring the potential use of rosiglitazone as a preventive and/or therapeutic agent for NMSC is an interesting issue with clinical importance. However, more researches are required to confirm the findings of the present study.

## Abbreviations

ICD-9-CM: *International Classification of Diseases, Ninth Revision, Clinical Modification*
IGF1: Insulin-like growth factor 1
NHI: National Health Insurance
NMSC: Non-melanoma skin cancer
PPAR: Peroxisome proliferator-activator receptors
